# Carotid Artery Segmentation in Ultrasound Images and Measurement of Intima-Media Thickness

**DOI:** 10.1155/2013/801962

**Published:** 2013-06-20

**Authors:** Vaishali Naik, R. S. Gamad, P. P. Bansod

**Affiliations:** Department of Electronics and Instrumentation Engineering, Shri Govindram Seksaria Institute of Technology and Science, Indore 23, Park Road, Indore 452003, India

## Abstract

*Background*. The segmentation of the common carotid artery (CCA) wall is imperative for the determination of the intima-media thickness (IMT) on B-mode ultrasound (US) images. The IMT is considered an important indicator in the evaluation of the risk for the development of atherosclerosis. In this paper, authors have discussed the relevance of measurements in clinical practices and the challenges that one has to face while approaching the segmentation of carotid artery on ultrasound images. The paper presents an overall review of commonly used methods for the CCA segmentation and IMT measurement along with the different performance metrics that have been proposed and used for performance validation. Summary and future directions are given in the conclusion.

## 1. Introduction

Cardiovascular disease (CVD) is one of the leading causes of deaths in the metropolitan cities. A recent survey by the World Health Organization revealed that up to 17.3 million people died from CVDs in 2008. Scientifically presaged the upcoming 2030, almost 23.3 million human deaths are resulting from CVDs [1]. CVD ailments are relative to atherosclerosis (arterial disease). Atherosclerosis is responsible for the thickening of the artery walls, and the IMT is used as a validated measure for the evaluation of atherosclerosis. Prominently, the increase in IMT jeopardizes the brain infarction or cardiac attack [2, 3].

Usually, the B-mode ultrasound scan of CCA in its common tract is used for the evaluation of the artery status and for the measurement of the IMT. Ultrasound methodology has manifest benefits of being real-time, noninvasive, low-cost, reliable, and absolutely safe for the patients. The essential drawbacks of this methodology are the B-mode image having low signal-to-noise ratio and ultrasounds are operator dependent [4]. Conventionally, the IMT is manually measured by the trained operator from the US scan images. This methodology is highly user dependent, time consuming, tedious, and infeasible in presence of large image databases [5, 6]. During the past 20 years, several computerized techniques have been developed for segmentation of CCA. These methods can be broadly classified into two categories: first category includes techniques that are completely automated, whereas second category includes those that require user interaction (semiautomated). This review will focus on the techniques that have been developed to perform CCA wall segmentation and IMT measurement in B-mode ultrasound images in both automated and semiautomated manners.

## 2. Clinical Significance of Vessel Wall Segmentation 

### 2.1. Common Carotid Artery Intima-Media Thickness

**Figure 1:**
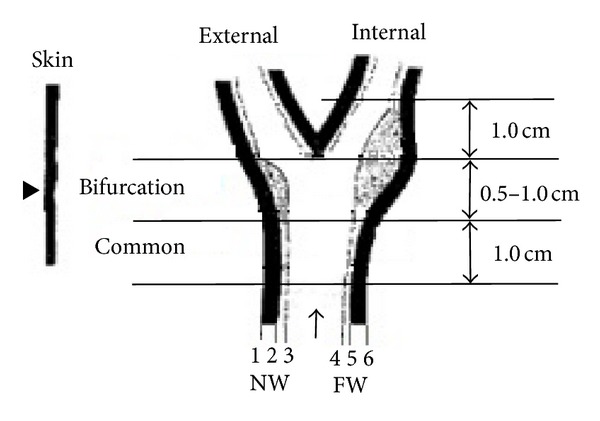
The carotid artery view with the interfaces: (1) periadventitia-adventitia (NW), (2) adventitia-media (NW), (3) intima-lumen (NW), (4) lumen-intima (FW), (5) media-adventitia (FW), and (6) adventitia-periadventitia (FW) [7].

The CCA longitudinal section is shown in Figure 1. It is characterized by a longitudinal tract (common carotid) that, after an enlargement (carotid sinus) containing a flow divider, bifurcates into two arteries, one internal and one external, on the basis of their position in relation to neck skin [7].

The bifurcation and the internal carotid artery (ICA) are more threatening to atherosclerosis, due to stronger hemodynamic stresses in the bifurcation and branching zones, but it is difficult to visualize the “double-line” pattern in these locations. So an IMT measurement in the CCA is preferred in the development of segmentation algorithms and in clinical practice [8].

**Figure 2:**
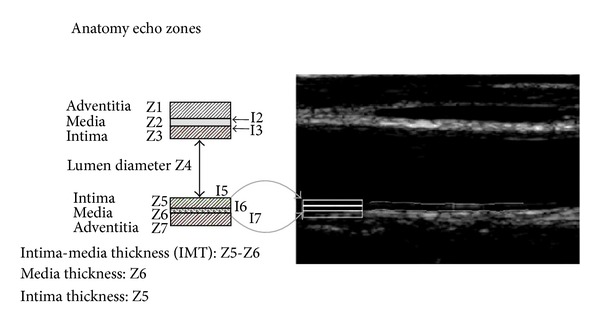
B-mode CCA image. The media layer thickness (MLT) is defined as the distance between the intima-media and the media-adventitia interface [11].

Classically, IMT is defined as a double-line pattern visualized by echotomography on both walls of the CCA in a longitudinal image. It consists of two parallel anatomical boundaries referred to as the lumen-intima and media-adventitia interfaces [9]. US waves are reflected differently by blood (vessel lumen) and wall layers because of their differences in density and elasticity. US waves are not reflected by vessel lumen and tunica media, thus allow detection of the lumen-intima (LI) and media-adventitia (MA) interfaces, as depicted in Figure 2 B-mode ultrasound CCA image [10]. 

### 2.2. Importance of Carotid Arterial Intima-Media Thickness

The increased IMT reflects early stages of atherosclerosis and cardiovascular risk. Higher blood pressure and changes in shear stress are the potential causes of intimal thickening. Changes in shear stress and blood pressure may cause a local delay in lumen transportation of potentially atherogenic particles, which favors the accumulation of particles in the arterial wall and consequent plaque formation [12]. It is found that type 1 diabetes is a significant risk factor for increased carotid IMT in children [13]. It is confirmed that the increase in IMT is directly associated with an increase risk of myocardial infarction and stroke in older adults without a history of cardiovascular disease [14]. The assessment of IMT in prediction of the degrees of atherosclerosis and the risk of stroke and CVDs has been demonstrated by a lot of studies [15–17]. It is found that obesity especially abdominal obesity in childhood and adolescence is closely related to IMT [18]. The studies confirmed that the increase of the IMT value above 0.9-1.0 mm is indicative of a significant increase of CVD risk when taking into account the population of healthy elderly [4, 7]. In the following sections, we will discuss the difficulties confronted in the intima and media detection and review the recent advancements in CCA segmentation.

## 3. Difficulties of Intima and Adventitia Detection

The difficulties encountered in detecting intima and adventitia layers are as follows [19]:

- presence of speckles in US image;
- the structure of IM complex or the intimal layer changes due to diseases such as atherosclerotic plaques;
- variation in echo characteristic on intima and adventitia on images with the variation in sonographic instrumentations.

## 4. Segmentation Techniques

Plethora of ultrasound-segmentation techniques have been reviewed in recent surveys by Noble and Boukerroui [29] and Molinari et al. [30]. Thereby authors are steadfast in reporting on recent techniques, used for the segmentation of the CCA on US images. These studies on the segmentation of the carotid artery boundaries include the application of dynamic programming, deformable snakes, hough transforms, and classification approaches to detect the carotid boundaries on longitudinally oriented images [27, 31–33]. For each methodology, the authors have described principles, performance, advantages, and limitations.  Table 1 gives and summarizes the technique that is mentioned below.

### 4.1. Dynamic Programming Techniques (DP)

DP technique is concisely used to solve optimization problems, where desired segmentation minimizes the cost function defined by the particular application. Local measurements of echo intensity, edge strength, and boundary continuity are included as weighted terms in a cost function. All possible set of spatially consecutive points forming a polyline is being considered, and favor is given to that which minimizes the cost function [34].

**Figure 3:**
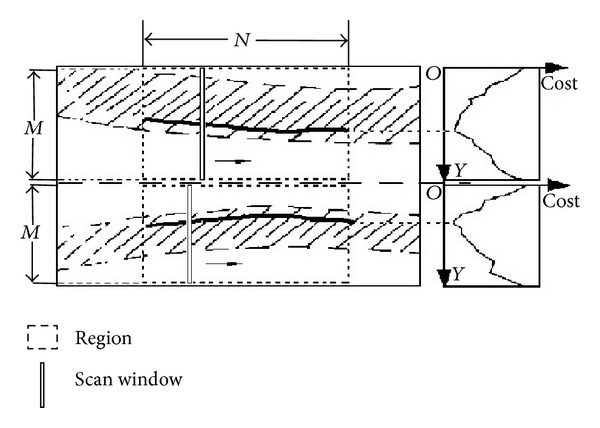
Detecting interfaces I2 and I7 in an artery image.

The polylines are represented as a vector:

(1) $$\begin{array}{c} P=\left(P_{1},P_{2},\ldots ,P_{i-1},P_{i},\ldots ,P_{N}\right), \end{array}$$

where *i* is the horizontal pixel position, *P*_*i*−1_ and *P*_*i*_ are neighbor points, and *N* is the horizontal length of the search region. A vertical search window of size *M* × 1 pixels is used to scan the boundary from left to right at *N* horizontal positions as shown in Figure 3. At scan position *i*, the boundary point, *P*_*i*_, in (1) can be any pixel in this window. The optimized connection is searched for each point in this window and the cost accumulated. At the end of the scanning, the optimal polyline is the one that minimizes the cost function:

(2) $$\begin{array}{c} C_{\text{Sum}}=\overset{N}{\underset{i=1}{\sum }}C\left(P_{i}\right). \end{array}$$

The local cost is a weighted sum of cost terms:

(3) $$\begin{array}{c} C\left(P_{i}\right)=W_{1}C_{1}\left(P_{i}\right)+W_{2}C_{2}\left(P_{i}\right)+W_{3}C_{3}\left(P_{i-1},P_{i}\right), \end{array}$$

where *W*_1_, *W*_2_, and *W*_3_ are weighting factors, *C*_1_, *C*_2_, and *C*_3_ are the echo intensity, intensity gradient, and boundary continuity cost terms, respectively. Based on DP techniques in 1994 Gustavsson et al. [28] introduced a procedure for automatic ultrasonic measurements of the carotid artery, and lumen diameter (LD) and IMT were computed. Intermethod (auto versus manual) variability as well as inter- and intraobserver variability was studied by computing the conventional coefficient of variation (CV). A major advantage of this methodology was complete automation and low computational complexity, thus suitable for clinical purposes. This method requires interactive tools for manual tracing in order to correct the remaining detection errors. The major limitation of this technique is the need for training of the system. In 1997, Gustavsson et al. [34] have compared four algorithms: the dynamic programming, the maximum gradient, the model-based, and the matched filter algorithm and confirmed that the DP algorithm provides superior performance in terms of accuracy and robustness. In 2008 Liu et al. [35] proposed a segmentation method in which the energy definition of active contour model was used and DP was employed to search the shortest path. To reduce the effects of speckle noise, anisotropic diffusion method was adopted. It is advantageous as it requires less manual input. Holdfeldt et al. [36] proposed a method based on DP for boundary detection in ultrasound image sequences. According to the author, this method gives favorable results on both synthetic and real ultrasound data. Cheng and Jiang [37] proposed a novel dual dynamic programming (DDP) technique that detected intimal and adventitial layers of the CCA of the B-mode US images. In this, the robustness against the speckles was increased by embedding the anatomical knowledge into its structure. Therefore, the researcher reported that the DDP technique achieved a detection performance comparable to manual segmentation.

### 4.2. Hough Transform (HT)

HT technique used to detect straight lines. A straight line at a distance *s* and orientation **θ** can be represented by

(4) $$\begin{array}{c} s=x\cos\theta +y\sin\theta . \end{array}$$

The HT of this line is just a point in the (*s*, *θ*) plane; that is all the points on this line map into a single point. This fact is utilized to detect straight lines in a given set of boundary points. If given boundary points are (*x*_*i*_, *y*_*i*_), *i* = 1,…, *N* for some selected quantized values of parameter *s* and *θ*, map each (*x*_*i*_, *y*_*i*_) into the (*s*, *θ*) space and count *C*(*s*, *θ*), the number of edge points that map into the location (*s*, *θ*); that is, set

(5) C(sk,θ1)=C(sk,θ1)+1if xicos⁡θ+yisinθ=sk for  θ=θi  .

Then the local maxima of *C*(*s*, *θ*) give the different straight line segments through the edge points. Generalized HT can be used to detect curves other than the straight lines [38]. Segmentation algorithm based on the HT was demonstrated by Golemati et al. [39] in 2007 to segment both longitudinal and transverse images. The HT is effective in detecting lines (longitudinal images) or circles (transverse images), but it may fail in detecting curved vessels. In 2008, Stoitsis et al. [40] proposed the HT-initialized active contour methodology. In 2010, Petroudi et al. [41] proposed a fully automated method that was proposed for the delineation of the intima-media complex (IMC). In this technique after speckle removal and HT used for boundary detection followed by image normalization, the corresponding results were used to provide the initial statistical information needed for a Markov random field (MRF). In 2011, Matsakou et al. [42] proposed a method in which an HT-based methodology was used for the definition of the initial snake followed by a gradient vector flow (GVF) snake deformation for the final contour detection. The author reported that the sensitivity, specificity, and accuracy were 0.97, 0.99 and 0.98, respectively, for both diastolic and systolic cases. Recently Xu et al. [21] proposed a segmentation method using HT and dual snake model; two contours are initialized from line segments generated by HT. Author admits that the technique is not suitable for irregular boundaries and decimate minor details.

### 4.3. Nakagami Mixture Modelling

In 2009, Destrempes et al. [25] introduced a segmentation technique based on Nakagami mixture modeling and stochastic optimization. The echogenicity of the region of interest (ROI) comprising the intima-media layers, the lumen, and the adventitia in an ultrasonic B-mode image is modeled by a mixture of three Nakagami distributions. In a first step the expectation maximization (EM) algorithm was used to compute the maximum A posterior (MAP) estimator of the proposed model, then computes the optimal segmentation based on the estimated distributions as well as a statistical prior for disease-free IMT using a variant of the exploration/selection (ES) algorithm. This method requires minimal manual initialization. Destrempes et al. [43] proposed a method for segmentation of plaques in sequences of ultrasound B-mode images of carotid arteries based on motion estimation and Nakagami distributions. In it, a local geometrical smoothness constraint and an original spatiotemporal cohesion constraint were incorporated, envisaging the segmented plaque based on motion field estimation. In 2011, Destrempes et al. [23] proposed a method for the segmentation of plaques in the sequence of ultrasound B-mode images of carotid arteries based on motion estimation and a Bayesian model. Authors have reported that the algorithm was not sensitive to the degree of stenosis or calcification.

### 4.4. Active Contour

The basic concept of active contour model is to fit a contour to local image information, for example, gradient. There exist several implementations of this basic idea such as snakes [44], discrete dynamic contour model [45], and level sets [46]. Based on the involved feature image, they can be categorized as edge based [47], region based [48, 49], and higher level knowledge based [50, 51]. Several studies have adopted the traditional snake model as proposed by Williams and Shah [52]. Snakes are also called active parametric contours, which have been widely used in medical image segmentation. The major limitations of this method are sensitive to noise, depends on the initial contour that is provided by the user, need for optimization of the parameters. In 2008, Moursi and El-Sakka [53] proposed an active contour-based segmentation technique, in which user only requires to place seed points in the ROI with the aim of reducing user interaction. Author admitted that the computational time depends on the size of the carotid artery and the location of the seed point. In 2010, Bastida-Jumilla et al. [54] used geodesic active contours for IMC detection. In 2011, Petroudi et al. [24] proposed the fully automated segmentation algorithm based on active contours, and active contours without edges were proposed in which anatomical information was incorporated to achieve accurate segmentation. The segmented regions were used to automatically achieve image normalization, which is followed by speckle removal. The resulting smoothed LI boundary combined with anatomical information provides an excellent initialization for parametric active contours that provide the final IMC segmentation. No information about an inter- and intraobserver variability and its effect on segmentations was given by author.

### 4.5. Edge Detection and Gradient-Based Techniques

The edge detection methods could detect the variation of gray levels, but it is sensitive to noise and may suffer from the focusing artifact. In 2001, Liguori et al. [7] proposed the segmentation technique based on an edge detection, in which image gradient was used. In this method, for each column of the image the gradient of the intensity profile has been computed. It was assumed that pixels belonging to lumen were black and that the carotid wall layers originate with gradient transitions. It is a semiautomatic method; ROI is selected by the user. The pattern recognition, edge detection (PRED) algorithm, and the measurement algorithm were used for carotid IMT measurement. Its main task is to find out all the pixels belonging to the two required interfaces (LI and MA) for each wall. The measured intensity gradient was different from the theoretical one, due to noise. In order to reduce the effect of noise, a statistical thresholding was adopted before computing the image gradient. Robustness of the edge detection algorithm had been evaluated with respect to the ROI. The gradient-based segmentation mainly suffers from the problem of superimposed noise, which precludes a proper individuation of the LI and MA transitions. In 2008, Faita et al. [26] proposed a method in which the gradient performance was improved by the use of a first-order absolute moment edge operator (FOAM) and a pattern recognition approach. The overall performance of this methodology was very high: IMT measurement error was equal to 10.0 ± 38.0 *μ*m. Moreover, FOAM operator and intelligent procedure determines maxima, ensuring a good robustness to noise. As the technique is real-time, it suits well to clinical application. It is a semiautomatic technique. Recently, Mahmoud et al. [55] introduced a method, which employs a multistep gradient-based algorithm. This method principally uses intensity, intensity gradient, and interface continuity of pixels to determine the ultrasound interface. Author reported that this technique eliminates subjectivity associated with conventional manual tracing and semiautomated gradient methods that employ seed point selection.

### 4.6. Combined Approaches

Delsanto et al. [6] proposed a combined approach for classification and a snake-based segmentation to perform IMT measurement. Completely user-independent layer extraction based on signal analysis (CULEXsa) is a completely user independent algorithm for IMT measurement. Firstly ROI is identified automatically followed by gradient-based initial segmentation, and then active contour technique is used for segmentation refinement. For improving segmentation performance, Molinari et al. [56] have combined the three IMT segmentation methods: (i) signal processing approach, combined with snakes and fuzzy clustering, (ii) integrated approach based on seed and line detection, followed by probability-based connectivity and classification, and (iii) morphological approach and fused the resulting boundaries using a greedy method described by the “ball and basket” to minimize the system error. In 2010, Molinari et al. [57] proposed a completely automated layer extraction technique (named CALEXia). The IMT measurement error was equal to 0.87 ± 0.56 pixels (0.054 ± 0.035 mm). Author admitted that CALEXia showed limited performance in segmenting the LI interface. Meiburger et al. [58] introduced the Carotid Automated Double-Line Extraction System based on the Edge Flow (CADLES-EF). It is characterized and validated by comparing the output of the algorithm with CALEXia and CULEXsa. Validation was performed on a multi-institutional database of 300 longitudinal B-mode carotid images with normal and pathologic arteries. CADLES-EF showed an IMT bias of 0.043 ± 0.097 mm in comparison with CALEXia and CULEXsa that showed 0.134 ± 0.088 mm and 0.74 ± 0.092 mm, respectively. The system's Figure of Merit (FoM) showed an improvement when compared with CALEXia and CULEXsa, leading to values of 84.7%, 91.5%, while CADLES-EF performed the best with 94.8%. In 2011, Molinari et al. [59] proposed a method called CARES 3.0 (a patented technology). CARES 3.0 is completely automated and adopts an integrated approach for segmentation of carotid artery in the image frame. The FoM of CARES 3.0 was 97.4%. In 2012, Molinari et al. [22] proposed completely automated multiresolution edge snapper (named CAMES). In it carotid artery is recognized automatically using a scale-space and statistical classification in a multiresolution framework. Recently, Ilea et al. [20] proposed a fully automated segmentation and tracking of the intima-media thickness in ultrasound video sequences of the CCA. For the video tracking procedure, a spatially coherent algorithm is introduced, which prevents the tracking process from converging to wrong arterial interfaces. Author reported that method can deal with inconsistencies in the appearance of the IMC over the cardiac cycle.

## 5. Validation/Quantitative Performance Assessment

Validation experiments are necessary in order to quantify the performance of a segmentation method. Validation is usually performed using truth models such as phantom studies, animal model studies, simulation, comparing the automated segmentation method with manually obtained segmentations. Following are the most used performance metrics to validate IMT measurements and computer traced boundaries [30]:

- Mean absolute distance (MAD),
- Hausdorff distance (HD),
- Polyline distance metric (PDM),
- Percent statistic test,
- Reproducibility of manual procedures. It is assessed by calculating intraoperator and interoperator variability using either of CV, MAD, HD, regression analysis, and Bland-Altman statistics,
- Manual and computer-measured IMT (intermethod). Comparison between the two sets was done using correlation or Bland-Altman plot.

## 6. Conclusion

This paper reports an extensive review of ultrasound carotid artery IMT segmentation techniques. Active contours, dynamic programming, and integrated approaches have been presented to segment the carotid wall and trace the boundaries of the LI and MA interfaces. None of the existing techniques were overwhelmingly good in all aspects. Characterization and validation studies will be required in order to carefully assess the effect of such variability on segmentation performance. Finally, we recognize that in the future, more work is likely to be done in segmentation based on adaptive segmentation for determination of IMT in ultrasound images of CCA with high IMT measurement accuracy, robustness, automation and reducing processing time. 

As in the case of fully automatic techniques, detection is not reliable, since it may detect the jugular veins edges. In addition performance of semiautomatic segmentation techniques is better than fully automatic segmentation techniques. Therefore, in future we will develop techniques in which human operator will select an ROI manually, and methodology will be based on adaptive segmentation with the aim of high accuracy, great robustness, and with reducing processing time. The proposed accuracy of detection of IMT algorithm falls within the inter- and intraobserver variability for the manual determination.

## Figures and Tables

**Table 1:** Overview of recent CCA IMC segmentation techniques for ultrasound imaging.

Name, year, and ref. no.	Common carotid artery IMT segmentation technique	Advantages & limitations of the methods	Selection method of ROI (SA/FA)	Performance metric	Processing time/frame *T*_avg_	IMT error (mm)	*N*
Ilea et al., 2013 [20]	Model-based approach, video tracking procedure: spatially coherent algorithm	Advantages: Method can deal with inconsistencies in the appearance of the IMC over the cardiac cycle. Robustness with respect to data captured under different imaging conditions.	FA	MAD	80 sec (8 sec in 1st frame & 72 sec in tracking 28 frames)	(−0.007) ± 0.176	40*
Xu et al., 2012, [21]	Hough transform and dual snake model	Advantages: It is less likely to be affected by noise, compensates the holes or missing boundaries, and can estimate the missing LI interface boundary. Limitations: The method would work fine for early thickening of IMC but fails for irregular boundaries in the presence of plaques and eliminates minor details.	SA	MAD	0.465 sec	0.02 ± 0.03	50
Molinari et al., 2012, [22]	Multiresolution edge snapper	Advantages: Complete automation, robustness to noise, and real-time computation. Limitations: Robustness with respect to noise, but the LI/MA representation is less accurate.	FA	MAD	Less than 15 sec	0.078 ± 0.112	365
Destrempes et al., 2011, [23]	Nakagami distributions, Bayesian model	Advantages: Robust to a reasonable variability in the initialization, lowest tracing error for LI & MA, method is not sensitive to the degree of stenosis or calcification. Limitations: Depends on initial segmentation	SA	MAD HD	38 sec	—	8988
Petroudi et al., 2011, [24]	Active contours & active contours without edges	Advantages: Fully automated, fast, does not require any user interaction, and works well for noisy images.	FA	MAD	—	0.09 ± 0.10	30
Destrempes et al., 2009, [25]	Nakagami distributions, stochastic optimization	Advantages: Reasonable average computation time, robust to the estimation procedures. Limitations: Method suitable for healthy arteries, extensive tuning & training, so computational cost is high, for different scanner requires retraining & retuning.	SA	MAD HD	24 sec	—	7283
Faita et al., 2008, [26]	First-order absolute moment edge operator	Advantages: Suited for fast real-time implementation, operator can have immediate feedback on the quality of the images. Limitations: Depends on ROI selection.	SA	MAD	—	0.001 ± 0.035	150
Liang et al., 2000, [27]	Multiscale dynamic programming	Advantages: No initial human setting, capable of processing images of different quality, ambiguous cases user can intervene, and reduced interobserver variability. Limitations: Training required, for different scanner retraining needed, searched LI & MA interfaces may cross each other.	FA	MAD	0.7 min	0.042 ± 0.02	50
Gustavsson et al., 1994, [28]	Dynamic programming	Advantages: Fully automated, low computational complexity; suitable for clinical purposes, human correction allowed. Limitations: Initial human setting & training required, fails for slanting IMC with weak boundary.	SA	MAD	—	0.03 ± 0.032	22

*N*: number of images/cases, SA: semiautomated, FA: fully automated, SD: standard deviation, HD: Hausdorff distance, MAD: mean absolute distance, *video sequences, *T*_avg_: average processing time/frame or image.
